# Role of Surgery in locally advanced prostate cancer

**DOI:** 10.12669/pjms.313.7103

**Published:** 2015

**Authors:** Syed Muhammad Nazim, Farhat Abbas

**Affiliations:** 1 Syed Muhammad Nazim, Section of Urology, Department of surgery, The Aga Khan University, Karachi Pakistan; 2 Farhat Abbas, Section of Urology, Department of surgery, The Aga Khan University, Karachi Pakistan

**Keywords:** Prostate cancer, Locally advanced, Radical prostatectomy, Surgery

## Abstract

A significant proportion of patients present with locally advanced prostate cancer with inherent higher risk of local recurrence and systemic relapse after initial treatment. Recent literature favors combination of radiation therapy and extended hormonal therapy for this subset of patients. The effectiveness of Radical prostatectomy (RP) alone for locally advanced prostate cancer is controversial and is a focus of debate. However, it can decrease the tumor burden and allows the accurate and precise pathological staging with the need for subsequent treatment. Comparison of RP and other treatment modalities is difficult and incorrect because of inherent selection bias. RP as a part of multi-modality treatment (with neo-adjuvant hormonal therapy (HT) and with adjuvant or salvage radiation therapy (RT)/HT) can provide long term overall and cancer specific survival. Surgical treatment (such as transurethral resection (TUR), JJ stenting and percutaneous nephrostomy (PCN) placement etc.) can also provide symptomatic improvement and palliative care to improve quality of life of patients with advanced prostate cancer.

**Sources of data/study selection:**

The articles published between years 1998-2014 were searched on electronic databases Pubmed, Science direct, Google scholar and Embase and used for preparation of this review.

## INTRODUCTION

Prostate cancer is the most common non cutaneous malignancy in the western world.^1^ Since 1980’s, radical prostatectomy is a treatment for clinically localized prostate cancer with a life expectancy of > 10 years and has certainly passed the test of time.^1^ While the average incidence of prostate cancer [Age –standardized rate (ASR) per 100,000 men per year] is reported to be much lower for Asian men i.e. 7.2 compared to 85.6 in the USA and 59.3 in Europe,^2^,^3^ it is partly believed to be due to lack of screening, poor diagnostic facilities and lack of awareness, especially in resource constrained countries. Due to lack of national level cancer registry, the epidemiology of this disease is not correctly known in Pakistan.^4^ In resource constrained countries like Pakistan, with the reasons cited above, a significant proportion of patients present late with more advanced disease at the time of detection, this makes them unsuitable for potentially curative treatment options.

Locally advanced prostate cancer is the one extending beyond the prostate capsule with invasion of peri-capsular tissue, apex, bladder neck or seminal vesicles but without any lymph node involvement or distant metastasis (i.e. T3-T4, N0, M0 disease).^5^ Nearly 20-25% of cases present as locally advanced disease. These patients are at increased risk of prostate specific antigen (PSA) failure, metastatic progression and cancer specific death.^5^ This definition can be interchangeably used with the high risk prostate cancer.

### High risk prostate cancer

D’Amico defined 3 factors which pose high risk to prostate cancer. These include clinical stage ≥T2c, serum PSA ≥ 20 ng/ml or biopsy Gleason score of ≥8.^6^

### Surgical treatment options for locally advanced prostate cancer

The surgical treatment options include definitive treatment i.e. Radical prostatectomy (RP) with intent to treat and procedures to provide palliation and symptomatic relief.

### Predictors of success for Radical prostatectomy (RP) in locally advanced prostate cancer

RP is considered as a standard of care for organ confined disease but traditionally it has been discouraged as a definitive treatment option for locally advanced prostate cancer because of the concern of positive surgical margins, increased risk of lymph node metastasis, local and distant relapse and consequently death from prostate cancer.^7^ Hence for cT3 disease, the effectiveness of RP in providing local tumor control is a focus of debate and is highly controversial.^8^-^11^

European association of urology (EAU) guidelines now support the role and recommend RP to be an appropriate option for selected patients with small low volume T3 tumor, PSA <20 ng/ml, Gleason score < 8 and life expectancy of > 10 years.^12^

The available tools to pre-operatively define patients’ risk of having locally advanced disease are imaging studies like Magnetic resonance imaging (MRI), pre and post-operative nomograms, PSA velocity and it’s doubling time.^12^

Pelvic MRI is the primary imaging study for staging. The methods employed to improve staging accuracy include endo-rectal coil MRI which can detect extra prostatic extension > 1 mm with sensitivity of 70% and specificity of 95% and has 100% specificity for the involvement of seminal vesicles.^13^

### Advantage of RP

The advantages of surgical treatment i.e. RP in locally advanced prostate cancer are several. It can decrease the tumor burden and allows the accurate and precise pathological staging. It therefore, can identify the patients who are at high risk of recurrence and can be subsequently managed by other combination (adjuvant treatment) e.g. external beam radiation therapy (EBRT) for margin positive disease or seminal vesicle invasion or hormonal therapy (HT) for e.g. in case of positive lymph node.^7^ It has also shown longer and durable results in terms of cancer specific survival compared to other options.

### Technical aspects of RP in locally advanced prostate cancer

RP in locally advanced prostate cancer requires special expertise and includes removal of whole prostate gland en-bloc (b/w urethra and bladder) with good apical dissection, wide resection of neuro-vascular (NV) bundle and complete resection of both seminal vesicles.^14^ The incidence of urinary incontinence and impotence are therefore higher in this group as compared to early prostate cancer but with increased surgical experience, the functional outcome can be improved and morbidity can be minimized.^14^,^15^ For locally advanced prostate cancer, open RP is preferred over laparoscopic approach and it should be done in high volume centers.^16^

Pelvic lymphadenectomy in cT3 disease is indispensible because of higher risk of lymph node involvement. The reported incidence of lymph node involvement is between 27-41% in different series.^17^,^18^ Briganti et al. recommended extended lymph node dissection to be carried out for patients with locally advanced prostate cancer.^17^ Heindenreich et al. compared the progression free survival (PFS) in patients with standard vs. extended lymphadenectomy and found a 35% benefit in favor of the later.^18^

### RP as a monotherapy

The data on surgical management of locally advanced prostate cancer has not been investigated or systematically reviewed and no large scale randomized controlled trial (RCT) is available to show its superiority. Comparison of RP with other treatment modalities for locally advanced prostate cancer is difficult and may not be correct because of heterogeneous group of patients and inherent selection bias of good prognosis patients in favor of surgery.^16^,^19^ A few studies have shown promising results of RP for locally advanced ≥cT3 disease. The oncological outcome and factors involved in prognosis of patients with locally advanced prostate cancer in different studies are presented in Table-I.

**Table-I:** Outcome and survival of Radical prostatectomy (RP) for locally advanced (≥cT3) prostate cancer.

Study	Setting/Country	Year	Patient’s (N)	Median follow up (Months)	Outcome assessed at (years)	BPFS (%)	CPFS (%)	CSS (%)	OS (%)	Predictive Prognostic factor identified
Hsu CY et al.^10^	Erasmus Medical Centre, Netherlands	2010	164	100 months	(5)	50.4	79.7	93.4	87.1	Tumor grade, margin and node status in CPFS.
					(10)	43.0	68.7	80.3	67.2	Grade, Nodal status and Pre- operative PSA in BFPS
					(15)	38.3	63.5	66.3	37.4	
Xylinas et al.^11^	GAU EST, France	2009	100	69 months	(5)	45	--	90	--	Gleason score >7, Pathological stage, Positive surgical margin and lymph node in cancer recurrence
Loeb et al.^15^	George Town University School of Medicine, US	2007	288	88 months	(7)	--	39	92	91	Pathological stage in biochemical progression
					(10)	--	35	88	74	
Freedland et al.^9^	John Hopkins Hospital, US	2007	58	156 months	(5)	62	90	98	--	Lymph node metastasis in cancer death
					(10)	49	80	91	--	
					(15)	49	73	84	--	
Gontero et al.^21^	Italy	2007	51	48 months	(7)	--	--	90.2	76.7	-------------------
Carver et al.^8^	Memorial Sloan Kattering, US	2006	176	76 months	(5)	48	86	94	--	Gleason score, Pre- treatment PSA, Year of surgery in biochemical progression
					(10)	44	76	85	--	
Ward et al.^14^	Mayo Clinic, US	2005	842	123 months	(5)	58	85	95	90	Pathological grade, Ploidy and Margin status
					(10)	43	73	90	76	
					(15)	38	67	79	53	
Van den Ouden et al.^19^	Netherlands	1998	83	52 months	(5)	29	59	85	75	Poorly differentiated tumor
					(10)	--	31	72	60	

BPFS = Biochemical progression free survival, CPFS = clinical progression free survival, CSS = Cancer specific survival, OSS = Overall survival.

In a multi-centre, non randomized 2 staged study (EORTC 30001), RP was done in clinical stage T3 patients with good prognosis factors (Age < 70 years, PSA ≤ 20 ng/ml, Biopsy Gleason score ≤ 7, Performance status 0-1 and Unilateral cT3a disease).^7^ The authors concluded that RP with extensive resection can be beneficial as a monotherapy for T3aN0M0 patients.

Van poppel et al.^20^ in their study determined the efficacy of RP monotherapy in men with clinically T3 disease. They mainly included patients with PSA values <10 ng/ml without involvement of seminal vesicles or lymph nodes and showed a 5 year biochemical recurrence free survival to be > 60%.

Gontero et al. in a single institution study showed that RP is technically feasible in any clinical ‘T’ extension up to M1a disease with acceptable morbidity.^21^ There were 51 patients in their study who had advanced disease compared to 152 patients with organ confined disease. This study showed no significant difference in terms of surgical morbidity except for the blood transfusion, operative time and lymphocele formation which were higher in advanced stage group. The 7 year overall and cancer specific survival rates were 77% and 90% in advanced group vs. 88% and 99% in organ confined groups respectively.^21^ They also proposed that a possible advantage of surgery will be debulking the disease and therefore prevention of complications related to local cancer progression.

Hsu et al. in their study attempted to determine the prognostic factors for advanced prostate cancer after RP in cT3 patients in terms of Biochemical progression free survival (BPFS), Clinical progression free survival (CPFS), Cancer specific survival (CSS) and Overall survival (OS) after 10 years. On multivariate analysis, they found that surgical tumor grade, margin and lymph node status were significant factors in clinical progression free survival (CPFS) and cancer specific survival (CSS) while surgical tumor grade, node status and pre-operative PSA levels were significant factors in biochemical progression free survival (BPFS).^10^

Xylinas et al. studied the role of RP for cT3 disease with the aim of disease control and showed that surgical experience is the main factor responsible to reduce peri-operative complications and produce better functional results.^22^ This meta-analysis shows that the biochemical progression free survival (BPFS) i.e. PSA <0.2ng/ml and 10 & 15 years survival ranges from 45-62%, 43-51% and 10-49% respectively. In fact these results were better than some of the series of EBRT alone or EBRT in combination of hormonal therapy (HT). The results however are not comparable because of lack of homogeneity.

Recently, a meta-analysis was published regarding the role of robot assisted radical prostatectomy for managing high risk prostate cancer showing comparable short term results to open prostatectomy in terms of its safety and functional outcome, however, the long term oncological data is still awaited.^23^

### Multi modality treatment

For locally advanced prostate cancer, various studies have shown that a multi-modality treatment is needed of which surgery is only a part. Early adjuvant and late salvage radiation (EBRT) or hormonal therapy (HT) can be considered in patients with locally advanced prostate cancer.^12^

A group of urologists at Mayo clinic has long been advocating RP as the first line treatment in multimodality approach for cT3 disease. Ward and colleagues^14^ in a large retrospective study with a follow up of 15 years showed that 78% patients with pT3 disease received adjuvant and salvage treatment (HT, RT or both) following RP. They categorized RP as an important part of multimodality approach for cT3.

### Neo-adjuvant and adjuvant treatment to radical prostatectomy (RP)

Many strategies have been devised to prevent the recurrence and improve the patient outcome after RP by neo-adjuvant and adjuvant therapies.^24^

### Neo-adjuvant hormonal treatment

The aim of neo-adjuvant hormonal treatment is to shrink the tumor, reduce the chance of having a positive margin and to reduce both local recurrence and distant metastasis in intermediate and high risk patients.^25^ However, neo-adjuvant HT is not routinely recommended and its role in clinical cT3 prostate cancer is controversial. It is also blamed by some experts to cause increased operative difficulty.^26^

Many studies have shown the impact of short term (6 weeks - 4 months) neo-adjuvant HT (including complete androgen blockade) before RP. A decrease in post operative +ve surgical margins is consistently reported along with decrease in biochemical recurrence but no effect on global or cancer specific survival was observed.^24^-^26^

### Adjuvant treatment

Adjuvant treatment is defined as RT or HT given within 90 days after RP while salvage treatment is given post-operatively after 90 days.^14^

### Post operative adjuvant radiation therapy (RT)

This approach is proposed in patients with suspicion of residual tumor after surgery or with risk of local relapse such as positive surgical margins, extra-capsular extension or seminal vesicle involvement.

Two randomized studies compared RP alone with RP + adjuvant EBRT for locally advanced prostate cancer. In EORTC 22911, Bolla et al. compared RP alone (n= 503) versus post-operative 60 Gy adjuvant radiation therapy (EBRT) (n= 502) over a six week period in patients with positive surgical margins or pT3 disease. After a median follow up of 5 years, the biochemical progression free survival (BPFS) was higher in group who received EBRT (74% vs. 52.6%, p<0.001) but no improvement in metastasis free, cancer specific and overall survival was found. The overall tolerability was good with acceptable low toxicity.^27^ They recommended immediate RT to those patients who had multifocal +ve surgical margins and Gleason score of ≥7. They also interpreted that RT can delay the need for HT and therefore can postpone its adverse effects.

Another trial SWOG 8794 compared patients with RP alone (n=211) with patients who received RP + EBRT (n=214) with pathologically advanced prostate cancer (pT3). After a median follow up of 11.5 years, this trial showed that adjuvant post op EBRT significantly reduces the risk of PSA relapse (median PSA relapse-free survival 10.3 years for combination therapy compared to 3.1 years for RP alone, p<0.001), and disease recurrence (median disease recurrence- free survival, 13.8 years for combination therapy vs. 9.9 years for RP alone, p=0.001), however, this advantage was not translated in terms of difference in overall survival.^28^

### Adjuvant Hormonal therapy (HT) after RP

The use of adjuvant HT after node negative RP was studied at Mayo clinic. Siddiqui et al. in a retrospective study compared 580 patients who received adjuvant ADT with 1160 patients who were observed only. Although there was a significant difference in 10 year biochemical progression free survival (BPFS) (95% vs. 90%) and cancer specific survival (98% vs. 95%), no difference in overall survival (OS) was observed.^29^

Messing et al. randomized 98 patients with node positive disease after RP to either immediate androgen deprivation therapy i.e surgical or pharmacological castration) (n=47) versus observation only (n=51). The median follow up duration was 11.9 years. A significant increase in overall survival (64% vs. 45%) in favor of ADT and also improvement in PSA recurrence free survival (53% vs. 14%) disease free survival (60% vs. 25%) and prostate cancer specific survival (85% vs. 51%) was observed.^30^

It is concluded that all patients with advanced prostate cancer must be fully counseled and informed about the likelihood of multi-model approach after RP i.e. EBRT for positive surgical margin, extra capsular extension (ECE) or seminal vesicles (SV) invasion and HT in cases of lymph node involvement.^22^

### Salvage therapy after RP

Following RP, a PSA value >0.2 ng/ml (two consecutive rise) represents recurrent cancer.^31^ Salvage therapy is considered to improve the outcome of these patients but at the cost of adverse effects. The choice of salvage treatment depends upon location of tumor recurrence and aggressiveness of disease.^31^

### PSA relapse after RP

Following RP, the salvage treatment options are RT or HT (in the form of androgen deprivation therapy (ADT), anti-androgen monotherpy or combined androgen blockade (CAB).

Local recurrences after RP are best treated with salvage RT with 64-66 Gy to the pelvis at a rising PSA level (preferably < 0.5 ng/ml).^12^ Salvage RT is given when rising PSA is thought to be due to local disease recurrence such as late PSA relapse, Gleason < 7, slow PSA doubling time (PSADT) and positive surgical margins.^12^

In cases which demonstrate short PSADT and SV invasion, the chances of microscopic metastasis and systemic relapse are higher and therefore the appropriate option would be to combine RT with HT.^32^

### Comparison of combination therapy involving RP with non surgical approach

Akakura et al. showed in a randomized trial a comparison of RP + ADT with EBRT+ ADT and provided the evidence that at 10 years, the outcome of former group was better than the later with biochemical progression free survival (BFS) of 76.2% vs. 71.1%, clinical progression free survival (CPFS) of 83.5% vs. 66.1% cancer specific survival (CSS) 85.7% vs. 77.1% and overall survival (OS) 67.9% vs. 60.9%.^33^

Saito et al.^34^ compared the outcome difference in patients with locally advanced prostate cancer who were treated with RP + hormonal treatment (ADT) vs. combined RT + ADT vs. ADT alone, and found that RT + ADT or RP + ADT offers better overall survival than ADT alone.

In a study, data from Surveillance, Epidemiology and End result (SEER) were reviewed, analyzing 1093 patients with node –ve or node +ve cT4 prostate cancer. Only 72 patients (6.6%) underwent RP with or without adjuvant treatment. The final analysis showed increased overall survival (OS) in patients with surgical arm compared to those who received RT or HT alone and a survival comparable to ones who received RT + HT.^35^

### Palliative surgical treatment options in advanced prostate cancer

These options aim to provide palliation and symptomatic control to improve quality of life in patients with incurable disease.^16^

In the setting of radio-resistant prostate cancer, salvage radical prostatectomy is the most effective secondary curative treatment with good oncological outcome and acceptable morbidity. Due to the effects of ionizing radiation on tissues with consequent fibrosis and obliteration of anatomical planes for dissection, salvage RP causes higher risk of complications than primary RP.^36^

Obstructive uropathy develops in cases of aggressive tumors and therefore is associated with significant lower survival. Ureteral or bladder neck obstruction is either due to local extension of prostate cancer or lymph node metastasis with consequent azotemia.^37^ The surgical treatment options include placement of supra-pubic (S/P) catheter or transurethral resection (TURP) for bladder outlet obstruction and percutaneous nephrostomy tube (PCN) or Double J (JJ) stent placement in cases of upper tract obstruction. Salvage cysto-prostatectomy with urinary diversion can be used to palliate the debilitating morbidity caused by EBRT such as small capacity bladder with intractable hematuria and clot retention.

Surgical castration is a definitive hormonal therapy for metastatic prostate cancer patients and is more reliable, cost effective and guarantees continued androgen deprivation as compared to pharmacological castration.^16^

### Transurethral resection of prostate (TUR)

Palliative channel TUR can be a safe treatment option for patients with bladder outlet obstruction to improve urinary symptoms as it provides a wide channel to void,^38^ however, TUR alone can adversely affect the oncological outcome and can cause significant morbidity. These patients have higher incidence of distant metastasis, increased recurrence rate and lower survival especially for T3-T4 stage and moderately to poorly differentiated tumors.^39^ This is because TUR is associated with dissemination of tumor cells and breach of lympho vascular channels which promotes vascular spread with disease progression and consequently a worsened prognosis. This peri-operative dissemination of tumor cells has been proved by RT-PCR (Reverse transcription –polymerase chain reaction) for the detection of PSA mRNA.^40^

A population data based (SEER) study with over 29,000 men with prostate cancer had 2742 (9.3%) patients who underwent a TUR after diagnosis of cancer. The data supported the hypothesis that TUR carried out within a few months after needle biopsy based diagnosis of prostate cancer is associated with risk of local tumor progression and greater all cause mortality. This data also showed that subset of patients who underwent TUR had higher incidence of JJ stent placement (odd ratio 1.76), supra pubic cystostomy (odd ratio 1.9) & PCN placement (odd ration 2.46), all of which represent signs of local disease progression.^39^

Another concern is that normal anatomic landmarks may be obscured in patients with diffuse carcinoma resulting in a rigidly fixed prostatic fossa and bladder neck and distortion of trigone with consequent damage to these structures.

Crain et al. showed that palliative TUR can be a technically safe procedure for patients with locally advanced prostate cancer with low peri-operative morbidity, minimal blood loss and short hospitalization.^38^ It provided significant improvement in symptoms and urinary flow rate but compared to TUR done for a benign prostate (BPH), had a higher chances of failed trial without catheter (TWOC) and re-operation.

## CONCLUSION

RP forms an important part of multimodality approach to locally advanced prostate cancer and can provide better outcome (combined with adjuvant and salvage treatment if needed) than RT or HT alone or combination of RT and HT. Large volume prospective studies are required to confirm these findings. Other surgical treatment such as TURP, JJ stenting and PCN placement can provide palliative care to improve quality of life of patients with advanced prostate cancer.
